# Hemophagocytic Lymphohistiocytosis After Greenlight Laser Prostate Surgery

**DOI:** 10.7759/cureus.32167

**Published:** 2022-12-03

**Authors:** Subhash Chander, Sushant Chaudhary, Roopa Kumari, Julia Hennessey, Oltjon Albajrami

**Affiliations:** 1 Faculty of Medicine, Mount Sinai Icahn School of Medicine, New York, USA; 2 Internal Medicine, Saint Mary's Hospital, Waterbury, USA; 3 Pathology and Laboratory Medicine, Mount Sinai Morningside, New York, USA; 4 Pediatrics, American University of the Caribbean, Cupecoy, SXM; 5 Nephrology, Saint Mary’s Hospital, Waterbury, USA

**Keywords:** massive splenomegaly, adult t cell lymphoma, lymphoma, greenlight laser prostatectomy, hemophagocytic lymphohistiocytosis (hlh)

## Abstract

Hemophagocytic lymphohistiocytosis (HLH) is a life-threatening systemic inflammatory disease. Multiple risk factors have been defined for the manifestation of HLH. While infection remains the top risk factor, having multiple surgical procedures has also been suggested as a potential risk factor for HLH. Our patient presented with generalized weakness, weight loss, and fatigue after having a greenlight laser prostatectomy for benign prostate hypertrophy; the patient deteriorated rapidly and was found to fulfill the HLH 2004 and modified criteria. We believe this patient had a rare bone marrow disorder with a rare complicated clinical and laboratory presentation.

## Introduction

Hemophagocytic lymphohistiocytosis (HLH) was first described in 1939 by two pediatricians, Scott and Robb-Smith [1]. Since then, HLH has had multiple names, including macrophage activation syndrome and hyperferritinemic systemic inflammatory disorder. However, after 1991, the hemophagocytic lymphohistiocytosis name was proposed by the Histiocyte Society [2].

HLH is primarily a pediatric disease, and most patients are diagnosed between one and six months of age [3]. Many Japanese studies reported a 40% disease prevalence in adults as old as 70 years of age [4,5]. In addition, patients with underlying cancers, especially B- and T-cell lymphomas, are more susceptible to HLH [4].

Most HLH patients present with a continuous high-grade fever (>38.5°C) and enlarged lymphohematopoietic organs (i.e., liver and spleen). A few cases were also reported with altered mental status, delirium, and psychosis [6,7]. Nearly 80% of patients presented with anemia, thrombocytopenia, and deranged liver function. Moreover, reported in a few cases were lactate dehydrogenase (LDH) elevation, hypofibrinogenemia, and hyponatremia associated with inappropriate antidiuretic hormone secretion [8,9].

## Case presentation

A 79-year-old man presented to the emergency department (ED) with a four-week history of progressive weakness and a 50-pound weight loss in the previous six months. The patient lived on the east coast and endorsed working in his garden, running a business, and being active. He had recently been very unsteady with his feet and complained of excruciating and progressively worsening ‘lightning-like’ pain in his back after taking a few steps. As per the patient, the weakness and back pain started after having a greenlight laser prostate surgery six weeks prior. 

The patient reported no fever, night sweats, chills, rigor, lumps or bumps, cough, abdominal pain, arthralgia, urinary problems, diarrhea, or vomiting. His other past medical history is significant for coronary artery disease with stent placement, and heart failure with reduced ejection fraction status post-pacemaker placement. The patient had a history of hypothyroidism, hypertension, hyperlipidemia, and gastroesophageal reflux disease. The patient denied ever smoking or using alcohol. He denied any tick bites or rashes on the body.

In the emergency department (ED), the patient was awake, alert, and active; he was oriented to time, place, and person. The patient was afebrile, with a blood pressure of 101/69 mmHg, a heart rate of 111 bpm, and a respiratory rate of 33 bpm. Cardiac and respiratory exams were normal. The spleen was palpable on the abdominal exam. No spinal or paraspinal tenderness was appreciated. The straight leg test did not worsen the back pain. The patient had normal muscle bulk, tone, strength, and reflexes. He did not exhibit Romberg signs. The finger-to-nose test and shin-to-heal test were both normal.

**Figure 1:**
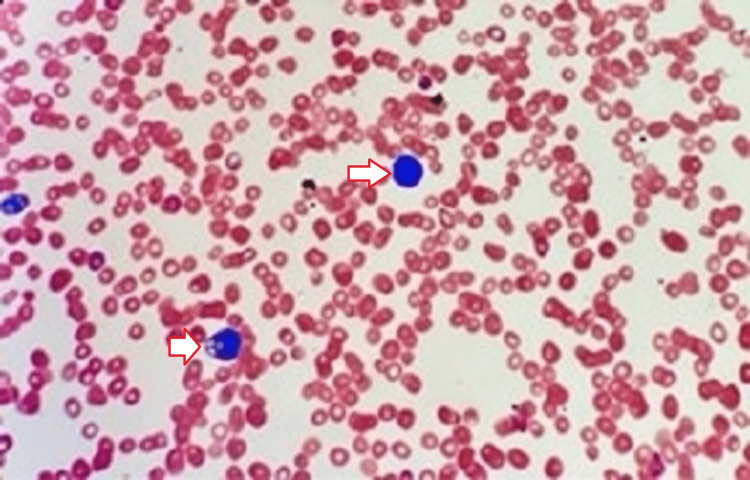
Peripheral smear of hemophagocytic lymphohistiocytosis.

**Figure 2:**
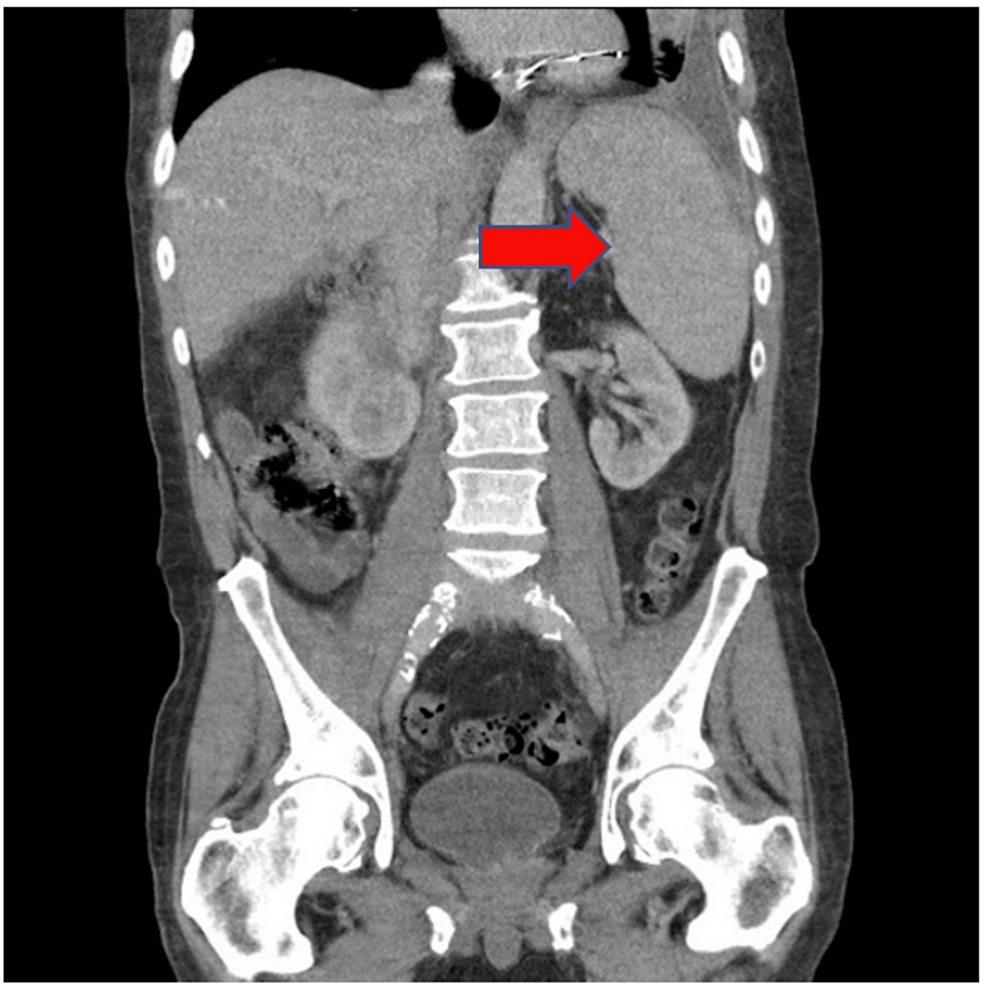
Computer tomography reporting massive splenomegaly.

Initial laboratory workup (Tables 1, 2) revealed hemoglobin of 10 g/dl, white blood cell count of 3.8k/ul, and platelet count of 87k/ul with a few atypical cells (Figure 1). His urine had many white cells, and urine leukocyte esterase was positive. The vitamin B12 level was 506 pg/ml and LDH was 832 U/L. Computer tomography of the chest, abdomen (Figure 2), and pelvis was done; previously known hydronephrosis of the right kidney was noted, as were moderate splenomegaly and atelectasis of the bilateral lungs.

**Table 1:** Laboratory investigations at the time of presentation. MCV: mean corpuscular volume, MCH: mean corpuscular hemoglobin, MCHC: mean corpuscular hemoglobin concentration, RDW: red cell distribution width.

Test name	Result	Range
White blood cell	3.8	4.0-10.5k/uL
Red blood cell	3.85	4.70-6.00 m/uL
Hemoglobin	10.1	13.5-18.0 g/dL
Hematocrit	31.7	42.0-54.0%
MCV	82.3	78.0-100.0 fl
MCH	25.9	27.0-31.0 pg
MCHC	31.3	32.0-36.0 g/dL
Platelets	87	150-450k/uL
RDW	20.5	11.5-14.0%
Seg neutrophils	73	%
Basophils	1	0-2%
Eosinophils	0	0-6%
Lymphocytes	7	20-48%
Monocytes	19	2-12%
Nucleated RBC	0	0-1/100 WBC
Neutrophils, absolute	3.9	1.5-7.0k/uL
Basophils, absolute	0	0.0-0.2k/uL
Eosinophils, absolute	0.1	0.0-0.6k/uL
Lymphocytes, absolute	0.6	1.0-5.0k/uL
Monocytes, absolute	1.1	0.1-1.2k/uL

**Table 2:** Additional labs. ALT: alanine transaminase, AST: aspartate aminotransferase, BUN: blood urea nitrogen, CK: creatinine kinase, PCR: polymerase chain reaction, SCL: scleroderma, TSH: thyroid stimulating hormone, LDH: lactate dehydrogenase, IL-6: interleukin-6.

Test name	Result	Normal range
Sodium	131	136-144 mEq/L
Potassium	4.2	3.5-5.1 mEq/L
Chloride	101	98-107 mEq/L
Bicarbonate	22	21-32 mmol/L
Anion gap	8	5-14 mEq/L
Glucose	105	74-118 mg/dL
BUN	27	7-25 mg/dL
Creatinine	1	0.7-1.2 mg/dL
Calcium	8.9	8.9-10.3 mg/dL
Magnesium	2.2	1.9-2.7 mg/dL
Phosphate	2.7	2.5-5.0 mg/dL
Albumin	3	3.5-5.0 g/dL
Bilirubin, total	2.2	0.3-1.0 mg/dL
Bilirubin, direct	0.7	0.0-0.2 mg/dL
Alkaline phosphatase	149	34-104 U/L
AST	92	13-39 U/L
ALT	37	7-52 U/L
Protein total	5.8	6.0-8.3 g/dL
Triglycerides	261	
LDH	832	140-271
Fibrinogen	350	145-415 mg/dL
Ferritin	6064.4	23.9-336.2 ng/mL
Haptoglobin	228	44-215 mg/dL
CK	12	30-223 U/L
SCL 70	94	<20 Units
Lactic acid	1.5	0.5-2.0 mmol/L
TSH	2.17	0.45-5.33 uIU/mL
Infectious disease labs		
Lab	Result	Reference range
Ehrlichia/anaplasma PCR	Negative	Negative
*Ehrlichia chaffeensis* PCR	Negative	Negative
*E. ewingii*/Cansis	Negative	Negative
*E. muris*-like	Negative	Negative
*Babesia microti* DNA PCR	Negative	Negative
B. duncani	Negative	Negative
B. divergens	Negative	Negative
IL-6	159.2	<6.4 pg/mL

His hospital course became complicated, and on day 2, the patient became confused and developed a new onset of a 101.2-degree fever. He was delirious overnight and had bladder incontinence. His blood and urine cultures continued to be sterile. The polymerase chain reaction for Anaplasma and Babesia was negative. Due to the patient’s acute inflammatory state, the hematologist felt the patient’s presentation was consistent with an infectious cause and recommended empiric treatment with doxycycline and the possibility of hemolysis, considering further elevated LDH (1626 U/L) and indirect bilirubin (1.6 mg/dl), even though haptoglobin (214 mg/dl) was within normal range.

On days 3-4, the patient continued to be delirious. He had an intermittent fever as high as 103.6 degrees with persistent tachycardia (102-113/min) and tachypnea (38-40/min). His exam continued to be unchanged, but his cytopenia worsened with hemoglobin of 8.7 gm/dl, white blood cells of 4.5k/ul, and a platelet count of 27k/ul. His serum triglycerides were 261, and interleukin 6 was 159.2 pg/ml. The antinuclear antibody was negative. The patient also tested negative for human immunodeficiency virus antibody, hepatitis B/C, Epstein-Barr, and cytomegalovirus. Nonetheless, given the rapid course of the disease and the high suspicion of a bone marrow disorder, the patient was sent for a bone marrow biopsy to obtain bone marrow cultures and cytology. At that time, the patient’s calculated H-score was 203 points, putting him at an 88% to 93% risk of HLH. The bone marrow biopsy was still in progress and was later reported as an inconclusive diagnosis. The patient’s family was made aware of the possible, highly likely diagnosis and was offered treatment with etoposide and dexamethasone; however, the family declined and instead opted for comfort care. 

Differential diagnosis

It is always a dilemma to differentiate the clinical picture due to sepsis, autoimmune disease, or malignancy associated with hemophagocytic lymphohistiocytosis. However, most HLH patients with febrile illnesses with multiorgan failure and abrupt onset of neurological manifestations. Nevertheless, signs and symptoms of HLH can occur in a variety of clinical presentations. Moreover, very high ferritin levels, an abnormal liver function test, and pancytopenia are crucial to differentiating HLH from other clinical problems.

Autoimmune Lymphoproliferative Syndrome (ALPS)

ALPS is a type of immune dysregulation syndrome that activates self-reactive lymphocytes. Majority of the patients present with hepatosplenomegaly, rash, and pancytopenia. However, ALPS does not present with multiorgan failure and high ferritin levels.

Infection and Sepsis

Infection and HLH share many common features, and due to that, it remains controversial to separate these two entities. Infection and sepsis, mostly not considered by lymphocyte activation and the dramatic increase in ferritin level, support the diagnosis of HLH.

Microangiopathic Hemolytic Anemia and Thrombocytopenia

Patient generally presents with fever, neurological manifestations, and renal failure. However, the key finding that differentiates HLH from these disorders is elevated ferritin, liver function abnormalities, and hemolytic anemia with a Coombs-positive status.

## Discussion

Hemophagocytic lymphohistiocytosis (HLH) is a very rare, life-threatening inflammatory disorder. The patient initially presents with high-grade fever associated with multiple organ failure, cytopenia, and neurological symptoms. The majority of the patients missed their initial diagnosis due to the variety of presentations. Patients may have experienced a prolonged hospitalization without a clear diagnosis before the possibility of HLH is raised.

Most HLH cases occur after an infectious or noninfectious trigger. Among infectious triggers, viral infections, including herpes (62%), Epstein-Barr virus (42%), and cytomegalovirus (9%), are noted to be more prevalent than other pathogens [4]. Only 9% of the cases reported have been due to bacterial infection, and among them, tuberculosis was reported in 50% of the cases [10]. However, surgery (cardiac surgery, colectomy, hepatic resection, splenectomy, colectomy, and postpartum) and severe burns are also noted as causes in some HLH cases [11,12].

The diagnosis of HLH is based on the corresponding clinical presentation in the setting of elevated inflammatory markers. A multidisciplinary approach with rapid evaluation for organ involvement and appropriate testing can provide better outcomes in HLH patients. The earlier differentiation is achieved, the more favorable the outcome. Ideally, the diagnosis of HLH should be based on the HLH diagnostic criteria published in 2004 [2,13]. 

The diagnosis of HLH can be made by either molecular diagnosis or via five of the following nine findings: (i) fever 38.5°C, (ii) splenomegaly, (iii) hemoglobin <9 g/dl and platelets <100k/ul, absolute neutrophil count (ANC) <1k/uL [14], (iv) hypertriglyceridemia fasting >265 mg/dl and/or hypofibrinogenemia (fibrinogen <150 mg/dl), (v) hemophagocytosis in bone marrow, spleen, lymph node, or liver, (vi) low or absent natural killer cell activity, (vii) ferritin >500 ng/ml, (viii) elevated soluble CD25 level, and (ix) elevated CXCL9 [15].

For patients with HLH, early diagnosis and urgent treatment measures are warranted for a liable outcome. Nonetheless, advanced and prompt treatment strategies have not fulfilled the desirable prognostic response [16], and many patients with HLH die rapidly after treatment due to multiorgan failure and sepsis [17]. Both anemia and thrombocytopenia are also present in >80% of the patient population [18]. CNS involvement can occur at any time of presentation and warrants an urgent indication for confirmation and initiation of medical treatment [19]. Additionally, treatment sometimes causes more harm than the disease itself, and mortality remains around 40%-50% [20]. The mortality rate is higher in the first week of treatment, likely due to complicated multiorgan failure. Increased age, thrombocytopenia, and comorbidities are associated with the worst prognostic factors [4].

## Conclusions

Our patient fulfilled the above criteria to establish the diagnosis of HLH due to presenting with fever, splenomegaly, Hgb <9 g/dl, platelets <100k/uL, ferritin of 6064 ng/ml, and triglycerides of 261 mg/dl. The calculated H-score of 203 points favors the diagnosis of HLH in this patient.

Infections, including viral and bacterial cases, are noted to remain the leading case of HLH. However, major surgeries like cardiac, abdominal laparotomy, and urological surgeries are also associated with the presentation of HLH in some cases. In this patient, the onset of HLH came after greenlight laser prostate surgery, which is unique among all reported surgical causes for HLH disease. Sudden deterioration complicated the course of treatment, and the family opted to forgo treatment and instead provide comfort care. The patient died the following day.
